# Designing a platform/adaptive randomised controlled trial for peripheral arterial disease (PAD) – The PAEDIS international platform trial development project

**DOI:** 10.3310/nihropenres.13556.1

**Published:** 2024-04-29

**Authors:** Athanasios Saratzis, PAEDIS development award consortium

**Affiliations:** 1 Cardiovascular Sciences, University of Leicester, Leicester, Leicestershire, LE3 9QP, UK

**Keywords:** Peripheral Artery Disease, PAD, randomised controlled trial, platform trial, claudication, chronic limb threatening ischaemia, CLTI

## Abstract

**Background:**

Peripheral artery disease (PAD) is a common health problem. There are several technologies, medications, and interventions that aim to improve or treat PAD in people with symptomatic disease. Most of these technologies, however, have been untested in high-quality randomised studies assessing effectiveness and their interactions remain unknown. We developed a proposed design for an international randomised controlled trial assessing multiple PAD treatments.

**Methods:**

Over the course of 11 months (2023) several workshops and reviews of the literature took place. More specific, the proposed platform trial was designed with 44 people with PAD and 112 experts from across the world, in five work packages. The most relevant PAD treatment with unproven effectiveness were identified and key trial components as well as success criteria were defined. With input from five clinical trials units, the final format of a potential platform PAD trial in primary and secondary care was then proposed for funding.

**Results:**

The proposed platform PAD randomised trial involved two major multi-arm multi-stage randomised studies, assessing PAD treatments in the community setting (1 ^st^ package) and then secondary care (2 ^nd^ package). The 1 ^st^ package involved people with claudication and the 2 ^nd^ package involves people with chronic limb threatening ischaemia (CLTI).

**Conclusions:**

A platform PAD trial involves many challenges in terms of both design and delivery. The proposed design involving both people with claudication and CLTI will hopefully act as a blueprint for future work in this area.

## Plain Language Summary

**Background:**

One in five people over 55 years of age have blockages in the arteries carrying blood to their legs. This is called peripheral artery disease or PAD. It can cause severe leg pain or skin and muscles of the legs dying due to limited blood supply. Doctors have been treating PAD using surgery for years. Keyhole artery surgery has recently been developed. Also, new medications are available for people with blocked arteries. Several new devices and medications are invented every year for PAD. Unfortunately, we don’t know whether these new medications and devices actually work. This is causing uncertainty when making treatment decisions, leads to unnecessary leg amputations, and deaths. Also, these new treatments might be costing society far more money than the older treatments. In this work, we designed the best possible research to assess all these new PAD treatments in the next few years.

**Design & methods:**

This project took place in 2023 in five different stages (called work packages), involving 44 patients, carers, and 112 experts from many countries and the NHS. We looked up all treatments and medications available for this condition. We then agreed on what would make our future research successful. After that, we set up groups of patients and experts to design the research. We agreed on the final design of this research.

**Results:**

The proposed study should involve people with claudication in the 1 ^st^ stage and people with leg gangrene in the 2 ^nd^ stage. We agreed to test treatment like exercise and open surgery vs. keyhole surgery. The best way to assess treatments is to look at which one leads to less deaths and leg amputations. The results of this work described in this article will act as a blueprint for future research in this area.

## Introduction

Peripheral arterial disease (PAD) is a common health problem, affecting 20% of those over 55 in Western countries. The prevalence of PAD has been steadily increasing in many countries, due to a sedentary lifestyle, increasing prevalence of obesity, and diabetes (all key risk-factors for PAD) ^1– 5^. At present, PAD is the commonest cause of major lower limb amputations and a leading cause of cardiovascular morbidity globally ^2, 5, 6^. In the UK, PAD is the most common pathology requiring specialist vascular care in a secondary (hospital) healthcare setting, as well as in the community (e.g. dressing changes for ulcers or repeated appointments for managing PAD-related complications). People diagnosed with PAD are very likely to develop other cardiovascular complications compare to age-matched controls, even when adjusted for relevant risk-factors ^2, 5– 8^. This is the case, even when PAD does not lead to lower limb symptoms (i.e. asymptomatic PAD). Around 5% of those with PAD will, however, also develop lower limb symptoms at some point in their lifetime. This group (with symptomatic PAD) typically present with intermittent claudication (IC) or Chronic Limb Threatening Ischaemia (CLTI); CLTI is limb and life threatening, requiring urgent revascularisation to ensure that patients do not die or lose their leg. Patients with claudication (IC) require exercise therapy and occasionally revascularisation to alleviate their symptoms. At the same time, all patients who have symptomatic PAD, including IC and CLTI, will medications and lifestyle changes in order to reduce cardiovascular risk and the further progression of their steno-occlusive arterial disease. This constitutes the backbone of PAD care and is necessary regardless of whether someone with PAD undergoes intervention (e.g. surgery) or not.

Most revascularisation procedures for PAD are endovascular i.e. minimally invasive (keyhole procedures). Several new endovascular technologies (e.g. stents and other adjuncts) and medications are regularly introduced in clinical care for patients who are undergoing lower limb revascularisation. There are several different such devices, and a plethora of treatment configurations is now possible. The clinical and cost-effectiveness of these devices and procedures, however, is unknown. Almost none have been assessed in high-quality randomised studies, with primary outcomes and designs that can assess clinically relevant endpoints such as amputation free survival or improvement in quality of life. As a result, their clinical and cost effectiveness in the short, medium or long term remain completely unknown. The rapid introduction of new PAD technologies and the fact that they can be used contemporaneously in many instances, alongside the vast heterogeneity of presentation of patients with PAD further complicate their assessment. Endovascular devices which work in various different ways are now available for almost all anatomical segments in the lower limb and large arteries.

Unfortunately, most of the above PAD technologies and new treatments are now used in routine NHS care without adequate scrutiny. This has been identified as a key research priority in a JLA Priority Setting Partnership (PSP) with the Vascular Society of Great Britain and Ireland (VSGBI). Further, patients in focus groups and surveys as part of this application and our ongoing NIHR-funded research are concerned about the lack of evidence to support decision-making when they are offered treatment to address their PAD. This includes people with both asymptomatic and symptomatic PAD, regardless of whether they are undergoing intervention or not. It also includes new technologies (e.g. endovascular devices) as well as more traditional treatments, such as various types of exercise programmes. Overall, the universal lack of high-quality randomised evidence assessing the effectiveness and long-term results of PAD technologies, medications, and exercise programmes is leading to more deaths, amputations, increased healthcare costs, and uncertainty regarding decision-making across the NHS and internationally. This can be addressed using a platform trial design, which will facilitate the assessment of many different and potentially overlapping PAD technologies in one research package. The best possible PAD related trial design/protocol would generate evidence rapidly, account for heterogeneity and variation in treatment-effects, assess multiple treatments at the same time, and take patients’ views into account. This work developed such a potential platform trial assessing new PAD technologies, aiming to improve patient care, end treatment uncertainty, and decrease healthcare costs, both in the NHS and globally. This can act as a model protocol for other randomised studies in the NHS and internationally.

We reviewed all PAD guidelines in 2022 to identify complex vascular trials, and currently available PAD treatments/medications. We recently published 3 international studies (52 centres, 3,289 patients) investigating new invasive treatments for patients with aorto-iliac or infrainguinal PAD ^7– 10^. We have identified the following:

•   Symptomatic PAD is the commonest arterial pathology requiring specialist treatment

•   There is variation in PAD medications and types of revascularisation offered to patients ^6, 11, 12^

•   Modern pharmacotherapies for PAD have not been assessed in high-quality trials

•   Most endovascular PAD treatments have not been tested in a randomised trial

•   Some new PAD treatments might be associated with increased mortality/amputation risk

•   Interactions between endovascular technologies and medications have never been assessed.

**Main aim:** Identify the optimal design and pathway for research delivery for a large-scale platform randomised controlled trial (RCT) assessing the clinical and cost-effectiveness of interventions for patients with symptomatic PAD. The **objectives per work package** are listed below.

**Work package 1 - evidence synthesis to identify:**

•   Ongoing and completed complex RCTs relating to any cardiovascular disease

•   Interventions to be assessed in the future platform trial (both interventional and pharmaceutical)

•   Comparators

•   Outcomes of interest, including relevant health states for economic modelling.

**Work package 2** - **set up lay and expert groups to guide research design**

•   Use our existing PAD lay groups to set up a new project-specific patient and public involvement panel

•   Institute an international group of professional stakeholders to lead trial design

**Work package 3** - define the trial’s **ideal characteristics** & key performance indicators ( **KPIs**) regarding:

- Screening, randomisation, and treatment allocation mechanisms
- Requirements needed to be met before an intervention is deemed appropriate to enter the trial
- Outcome assessments, treatment delivery assessment per arm, health economic data collection
- Cost-effective design and model structure, sustainability, and longevity, including use of existing resources (e.g. routinely collected data, existing cohort studies, National Vascular Registry integration)
- Implementation research and qualitative appraisal within the trial

**Work package 4** - gain **consensus** to design the research

- Finalise the PICO design of the ideal PAD platform trial
- Finalise KPIs to assess patient safety, research delivery success, and milestones
- Create a blueprint for collaboration between existing vascular registries, trials, and cohort studies in the NHS and abroad to establish recruitment and patient follow-up strategies
- Establish a mechanism via which arms will be added or removed in the platform trial
- Identify how interactions between different treatments will be assessed
- Create a vehicle for efficient and timely international dissemination alongside trial delivery.
- Consensus on appropriate core structure for economic modelling

**Work package 5** - finalise the funding application for the National Institute for Health and Care Research, by the end of November 2023.

## Methods

### Patient and public involvement

Patients were involved at all stages of this development process, including two formal patient co-applicants, who were part of the research team. Patients guided the research questions and guided all of the qualitative work in this development process. They chose outcomes of interest for the future potential study, outcomes, timings of assessment, format of recruitment methodology, inclusion or primary and community care pathways and duration of follow-up.

### Study and process design

A mixed methods approach was used in order to address the project objectives, in 5 separate work packages. The format and duration of each work package were proposed originally by our lay co-applicants and were further developed by our research team. Also, qualitative information from our recently completed NIHR Research for Patient Benefit (RfPB) CHABLIS study (ISRCTN registration: ISRCTN13202085) investigating barriers and enablers to provision of best medical or surgical therapy in chronic limb threatening were used. In CHABLIS ^13^ we interviewed 120 patients with advanced PAD (most with tissue loss or rest pain) treated in the NHS, to understand the pathways of treatment nationally, barriers to best medical therapy, barriers to receiving prompt intervention, and what they viewed as enablers to interacting with primary or secondary care.

The 5 work packages were subsequently further developed during a series of weekly meetings involving the core research team, including national and international representation across relevant specialties and healthcare professions involved in PAD care. Methodologists from 3 clinical trials units (CTUs; Edinburgh, Imperial, Leicester) with specific expertise in surgical trials and complex research delivery were involved. Further, the Vascular Society of Great Britain and Ireland (VSGBI), Royal College of Surgeons of England, British Society or Interventional Radiology (BSIR) were represented formally during these meetings to design the work packages. International vascular research networks were included at this stage i.e. the Research Collaborative for PAD (RCPAD - formal presence in 17 vascular centres across Europe) and Vascular and Endovascular Research Network (VERN - presence in 53 countries).

**Work package 1** - evidence synthesis to inform subsequent study design

The 1 ^st^ work package commenced immediately upon confirmation of funding in January 2023.

The MEDLINE, EMBASE, clinicaltrials.gov, WHO Trials Database and ISRCTN were searched systematically (since inception) using Medical Subject Headings (MeSH) terms relating to symptomatic PAD in order to identify all of the following:

1. Ongoing and completed complex RCTs in patients with vascular diseases
2. Interventions to be assessed in this clinical context
3. Comparators
4. Outcomes of interest

NHS Library resources were used. Abstracts were screened and reference lists searched to identify potential publications or online repository information regarding the aforementioned areas. An expert and lay summary of findings was prepared.

**Work package 2** - setting up lay and expert groups to guide research design

Alongside the systematic review and evidence synthesis, we set up the lay and expert groups for this research

### Lay groups

We recruited to a new project-specific patient and public involvement panel, including eight members from varied backgrounds. This group took part in our future workshops and focus groups. All lay participants received training online regarding how they can take part in focus groups and we answered all their questions.

### Expert groups

We instituted an international group of relevant professional stakeholders, including representation across specialties involved in PAD care as well as industry representatives. We recruited from the European Society for Vascular Surgery (ESVS), the Society for Vascular Surgery (America) and existing links with Australia and New Zealand. Industry partners from companies which manufacture products and therapies identified in work package 1 were invited. The British Society for Endovascular Therapy invited key endovascular industry partners. We advertised the project via the Society for Vascular Nursing, physiotherapy, podiatry, pharmacy, and orthopaedic networks in the UK, NHS, and abroad.

**Work package 3** - define the ideal characteristics and key performance indicators (KPIs) of a PAD platform trial 

This involved a one-day workshop with representation from our expert project-specific group, our lay co-applicants and those from the PPI group who wish to take part. The event was recorded and analysed. Results from work package 1 were sent to all participants beforehand.

The following areas were discussed, in order to reach consensus:

- **Participant identification pathways**
- **Screening mechanisms using existing care pathways and disease-specific registries**
- **Randomisation strategy**
- **Treatment allocation**
- **Treatment fidelity assessment**
- **Outcome assessment**
- **Health economic data collection**
- **Cost-effective design**
- **Longevity of the trial**

**Work package 4** - gain consensus to design the research

We agreed (online meetings of the core team and lay groups), on the following:

- Finalised the design of the ideal platform trial in this context (PICO)
- Finalised the KPIs to assess success research delivery success and milestones
- Established how eligible patients will be identified and approached efficiently based on unique regional and national characteristics
- Created a blueprint for collaboration between existing vascular registries, quality improvement programmes, and cohort studies in the NHS and abroad to streamline recruitment and patient follow-up in the platform trial.
- Established a mechanism via which new technologies made commercially available in this clinical context (revascularisation for CLTI) will be added in the platform trial in the future (ongoing evidence synthesis throughout the lifecycle of the trial)
- Identified how interactions between different interventions will be assessed.
- Identified success and failure criteria for each intervention relating to safety, clinical, and cost-effectiveness.
- Gathered consensus (patients and stakeholders) regarding the barriers and enablers of delivering the research on time and cost.
- Created a vehicle for efficient and timely international dissemination alongside the delivery of the trial.

**Work package 5** - finalised the trial protocol and funding application.

### Data collection & data analysis

All workshops, focus groups, and (where necessary) interviews were recorded and, if appropriate, transcribed. Thematic analysis was used to summarise the key findings of qualitative data.

## Results

### Evidence review (Results from work packages 1 and 2)

We performed three scoping literature reviews, 12 workshops (lay, industry & stakeholders’), meta-analyses on PAD technologies ^14, 15^, contributed to international guidelines ^11^, and updated a network meta-analysis on exercise therapy ^10^. Key findings:

-There are no ongoing complex PAD trials

-Medical and exercise therapy are the key constituents of conservative PAD care and interact with all invasive PAD technologies ^10, 16, 17^

-The recent BASIL2 ^18^ and BEST-CLI PAD RCTs ^19, 20^ did not assess most new invasive PAD technologies or their interactions ^19, 21^, despite having jointly costed £31 million

-Despite recent attempts ^22, 23^, there is no effectiveness-data regarding novel antithrombotics in PAD ^11^

-Home-based PAD exercise programmes have unproven cost-effectiveness ^24^.

### Design and research plan for the proposed complex PAD trial (Results from work packages 3–5)

Overall, we involved 44 lay participants, 81 healthcare professionals, representatives from four research funding bodies, and methodologists from five clinical trials units, (CTUs) who took part in the development-work Work Packages 3 to 5 and co-designed PAEDIS.

Our work proposed the following PICO design with regards to the proposed trial:

### Participants

People with symptomatic PAD>18 years (intermittent claudication or CLTI)

### Interventions

(selected as key PAD technologies needing effectiveness-testing in our reviews & multiple PPI/expert workshops):

-Home-based exercise therapy using the Motivating Structured Walking Activity in IC (MOSAIC) regime ^24, 25^

-Oral anticoagulation with Rivaroxaban 2.5mg & Aspirin 75mg (before surgery)

-Endovascular (minimally invasive) revascularisation of the common femoral artery

-Use of arterial stents when revascularising below the knee arteries (crural)

-Use of ultrasound-based surveillance after endovascular revascularisation

**Comparator**: Standards of NHS care (pragmatic design)

**Outcome** (primary): 1) Composite of death and/or major lower limb amputation (amputation free survival) over a minimum two year follow-up for those with CLTI & 2) Quality of life (EQ-5D-5L) for those with claudication. Our PPI and previous evidence strongly support a minimum 2-year follow-up. Secondary outcomes include mortality, various validated quality of life measures, cardiovascular events, re-admissions, amputations, and a health-economic analysis.

Our work led to the below project plan for the proposed trial:

### Design

**Figure 1.**
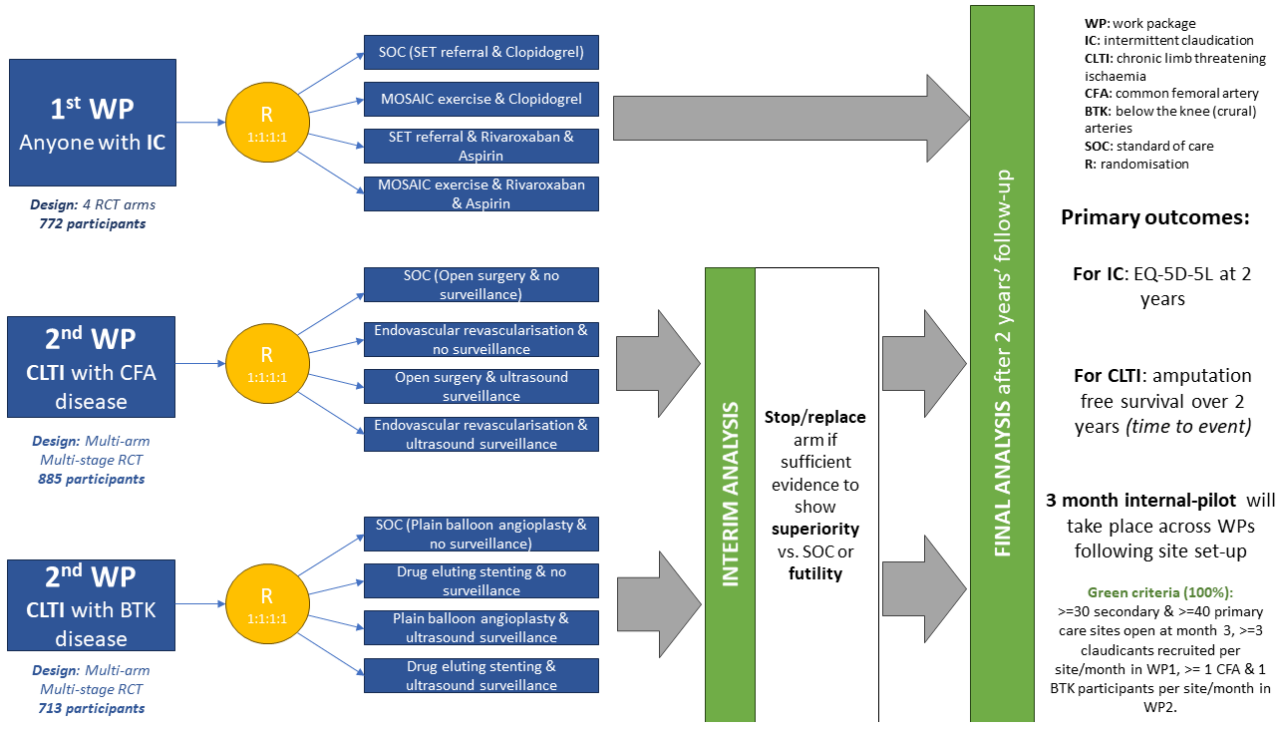
Final design of the proposed platform trial.

Platform multicentre prospective RCT. The design is summarised in the flowcharts ( Figure 1– Figure 3); it includes two work-packages, based on symptoms (claudication or CLTI) & anatomical segment treated.

**Figure 2.**
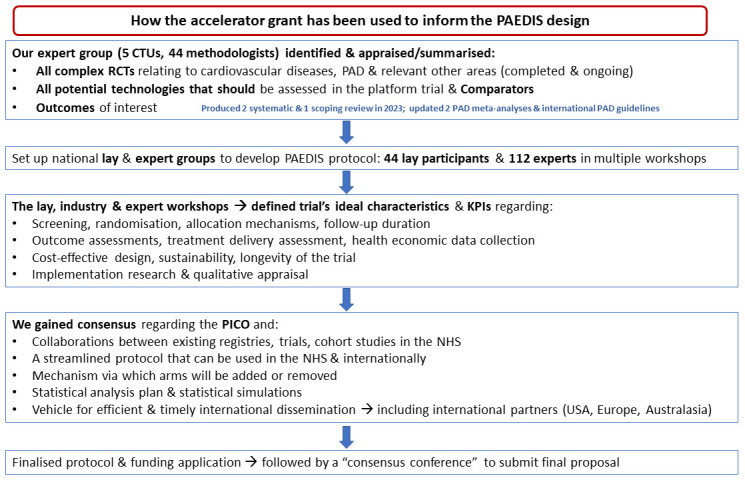
Work package of the development process during 2023 in order to finalise the design of the proposed platform trial.

**Figure 3.**
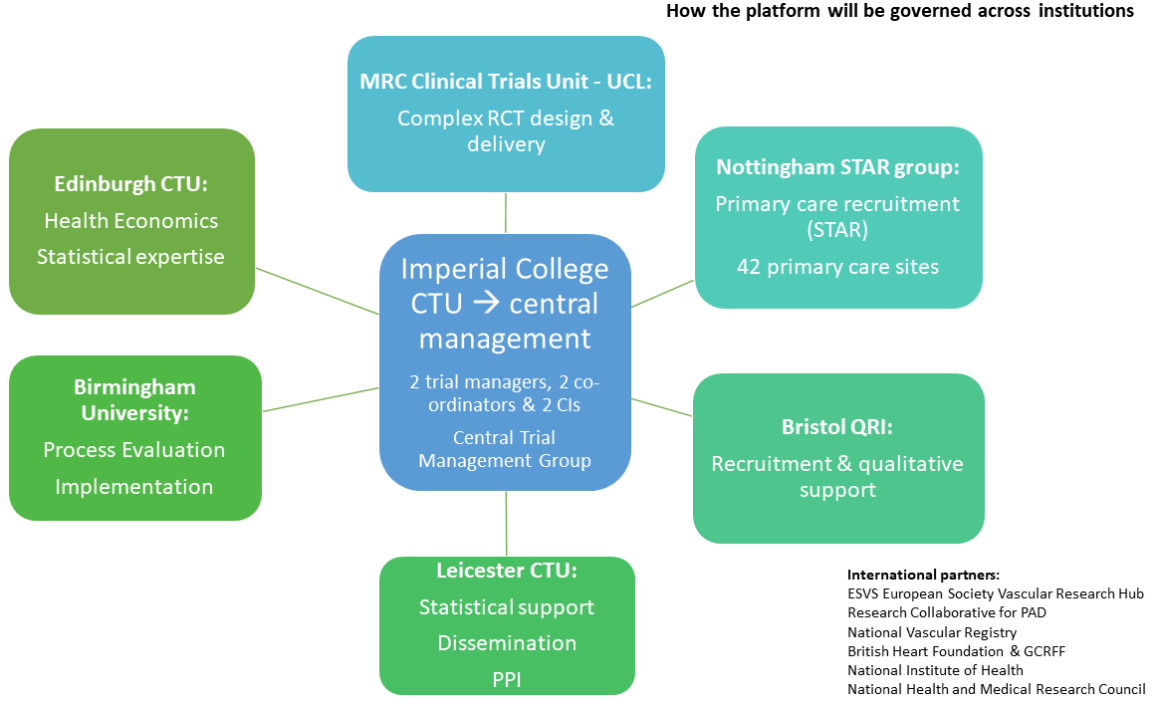
Proposed management plan for the institutions involved in the work.

**WP 1 (pre-surgery).** Four arm randomised (1:1:1:1) study involving people with claudication randomised to: 1) Referral for supervised exercise therapy (SET) & Clopidogrel (current NHS SoC), 2) MOSAIC exercise ^25^ & Clopidogrel, 3) SET referral & Rivaroxaban & Aspirin, 4) MOSAIC exercise & Rivaroxaban & Aspirin.

**WP 2 (surgery or post-surgery)**. Two multiarm-multistage (MAMS) randomised (1:1:1:1) studies involving people with CLTI, depending on which arterial segment is treated. Arms will be:

For those with common femoral artery disease: 1) Endovascular surgery with post-operative ultrasound surveillance, 2) endovascular surgery without surveillance, 3) open surgery with surveillance, 4) open surgery without surveillance (SoC).

For those with below the knee (crural) artery disease: 1) plain angioplasty with ultrasound surveillance, 2) plain angioplasty without surveillance (SoC), 3) primary stenting with surveillance, 4) primary stenting without surveillance. 

One interim analysis will take place in each WP2 component, to terminate or replace arms when there is strong evidence either in favour or against clinical effectiveness. Recruitment will continue during the interim. If results reveal an arm is to be terminated (lack of effectiveness vs. SoC), randomisation to that arm will be stopped, with participants allocated equally between remaining arms. If an arm is superior vs. SoC, recruitment to SoC will be stopped. The superior treatment will be the new comparator. Only if all arms are terminated will recruitment be stopped.

### Setting

Thirty NHS hospitals & 42 primary care sites (including the 5 NHS regions with highest amputation & diabetes rates). NIHR CRN and vascular research collaboratives who co-developed PAEDIS will support set-up. Four major funders (USA, Europe, Australia, New Zealand) co-designed PAEDIS to allow expansion to 6 European countries, USA, Australia, New Zealand via an international protocol.

For WP1, recruitment will be community-focussed; WP2 will mostly recruit in hospitals. A bespoke PAEDIS web-based toolkit and software management system developed by TCR Nottingham, who support GP practices’ RCT recruitment UK-wide, will be used for WP1, co-managed with the STAR team (Nottingham). Eligible patients will be identified at each practice using an automated system and then recruited centrally.

### Population, inclusion/exclusion criteria

Adult with symptomatic PAD, regardless of protected characteristics.

### Technologies assessed

Rivaroxaban with Aspirin; Home-based exercise therapy (MOSAIC); Endovascular revascularisation of common femoral artery disease; Endovascular revasularisation of crural arteries using primary stenting (drug eluting); Ultrasound surveillance after endovascular revascularisation for symptomatic PAD. All are efficacy-tested and used in the NHS already.

### Measurement of costs & outcomes

A prospectively planned economic evaluation will be conducted from an NHS and personal social services perspective, as per National Institute of Care Excellence (NICE) recommendations ^26^. Use of hospital and community services will be recorded using diaries/records. Quality-of-life will be assessed using EQ-5D-5L (alongside three other claudication questionnaires), converted to health-utility scores using the UK value set recommended by NICE. Unit costs will be based on manufacturers’ prices. Days lost from work and activities will be recorded and used in a secondary analysis. The extent and pattern of missing data will be assessed and appropriate methods employed. A state-transition decision model will be constructed to estimate costs and QALYs over the lifetime of the cohort. Outcomes or states of the model will be decided based on expert consultation and previous PAD economic evaluations. 

### Long-term follow up

Participants will be consented to allow data to be linked to routinely collected datasets across the NHS; we have collaborated with Health Data Research UK, British Heart Foundation, and National Vascular Registry (NVR).

### Sample size, recruitment

Sample sizes are calculated to provide 90% power at 2.5% significance level (one-sided). Multiple testing adjustment is applied to allow for the fact that, in each population each experimental treatment is compared to SoC twice. We adjust for multiplicity by setting the significance level for each trial arm at 1.25% (one-sided); 2.5% thus represents the total family-wise type I error-rate per treatment. 

***WP1*** utilises a multi-arm parallel group design. The primary outcome (EQ-5D-5L at 2 years) is continuous and normally distributed, with an estimated standard deviation of 0.2 ^27^. The study aims to detect a treatment effect of 0.074, found to be the minimum clinically important difference in the EQ5D-3L measure amongst people with similar characteristics ^28^. This was deemed appropriate by our PPI. The required sample size is 183 per treatment arm, or 732 in WP1, increased to 772 allowing for 5% dropout.

***WP2*** utilises a multi-arm multi-stage design deployed in parallel in the CFA & BTK populations. Within each parallel trial there will be a single interim analysis using asymmetrical non-binding O’Brien-Fleming efficacy and futility boundaries. The primary outcome is amputation free survival at 2 years. The 2-year probability of amputation or death among CFA & BTK patients is 0.53 based on the latest BASIL-2 & BEST-CLI RCTs and our cohort studies/meta-analyses for CLTI ^8, 14, 18, 19, 29– 34^. We aim to detect a 32% reduction in amputations/death in the arm receiving the more invasive treatment. This value was set by PPI participants as the magnitude of reduction they would require to make a more invasive treatment acceptable and accounting for delays to receive the randomised surgery and has been widely used in HTA-funded PAD research/trials (all BASIL trials). We anticipate recruiting 0.84 CFA and 0.38 BTK participants monthly based on BASIL-2, BASIL-3, BEST-CLI RCTs, and our current PAD research portfolio. The required sample sizes are calculated based on a constant hazard rate in each treatment arm and allow for expected accumulated events (partial follow-up). In the CFA component, a recruitment window of 25 months with an interim at 12 months from the start of recruitment would provide sufficient power in the absence of any dropouts or missing data. In the BTK component, a recruitment window of 45 months with an interim analysis at 32 months from the start of recruitment would be sufficient. These correspond to maximum sample sizes of 882 (CFA) and 718 (BTK). To allow for dropouts, missing data, and delays to receive surgery, we extend the recruitment window to 26 months (CFA) and 48 months (BTK), leading to a maximum sample size of 918 (CFA) and 766 (BTK). If recruitment to any treatment arm is stopped at the interim analysis stage, then the actual sample size required will be less than these maximums. As a result, the expected (mean) sample size is 885 (CFA) and 713 (BTK).

### Statistical analysis

In WP1 the primary outcome will be analysed using mixed effects linear regression adjusted for covariates including a fixed effect for baseline EQ-5D-5L and a random effect for site. In WP2, time to amputation/death will be analysed using mixed effects proportional hazards regression adjusted for a random effect for site. Roughly 4% of claudicants enrolled in WP1 may progress to CLTI and becoming eligible for WP2; we do not expect this to cause substantial bias. Sensitivity analyses will explore alternative analytical strategies.

## Discussion

This is the first, to our knowledge, systematic international attempt to design and finalise a protocol for a complex trial relating to assessing the effectiveness of novel PAD treatments, across society and healthcare settings. This work led to a proposed trial design which included treatments for PAD in community and secondary healthcare settings. Consensus was achieved via interaction with lay individuals internationally and key stakeholders. Unfortunately, the NIHR Health Technology Assessment Programme did not award funding in order to deliver the subsequent proposed trial. At the same time, the findings of this development work will benefit future researchers in many different ways. We identified all of the following:

1) Key areas of interest in PAD research, both to the public and researchers/clinicians
2) Vehicles and sample size/statistical analysis plans for delivery of complex PAD work
3) PAD technologies used in the NHS and other healthcare environments without effectiveness data.

Despite the fact that the eventual application to the NIHR in order to proceed with the delivery of the proposed trial design, was unsuccessful, this work still offers valuable insights into the ongoing and potential future research environment relating to PAD treatments.

More specifically, we identified a number of untested interventions already used in clinical care, despite lack of randomised effectiveness-level (clinical and cost) data. This is an issue internationally and not just in the NHS. These interventions include exercise regimes as well as invasive treatments across various anatomical areas, especially in the common femoral artery and crural (below knee arteries) arteries.

Further, this work identified a number of challenges or barriers relating to the delivery of such trials in the NHS as well as abroad. This was reflected in the feedback received from the NIHR when applying for subsequent funding; the overall cost of the application (exceeding £10 million) was felt to be excessive and not offer value for money. This calls for international trial collaborations amongst funding bodies, otherwise such ambitious research projects will never be delivered. The high cost of randomised studies has long been identified as a challenge in terms of delivering (and designing) these studies ^35^. At the same time, unless such large-scale pragmatic randomised studies are funded, either by public bodies and/or industry partners, we will never address queries relating to the effectiveness of new PAD technologies. In previous research, we have identified this major issue in the current PAD randomised literature ^36^.

Another important item to take into account regarding future steps and considerations, is the overall unanimous view of patients taking part in our groups that this work is important and their opinion(s) that randomisation is acceptable. All patients have supported this attempt to develop such a complex trial and found that both randomisation and takin part in multiple studies is not only of interest to them, but essential for future evidence generation. We did not encounter major concerns about the nature of these sub-studies and overall design by the patients. Their main input surrounded primary outcome measures, viewing death and amputations as the main issues, and participation in multiple sub-studies – viewed generally acceptable.

With regards to future steps, we propose that the sub-projects detailed in our research plan should be split into individual work packages. This will allow costs to be within the funders’ budget and provide value for money.

Our group also will share the qualitative data we have obtained in this process with any group willing to design a trial or similar research in these contexts.

## Conclusions

This work has developed a study design and protocol structure for a potential future platform/adaptive randomised controlled trial relating to peripheral artery disease. It can be used as a blueprint for future trials in this area.

## Data Availability

No data are associated with this article.

## Notes

**PAEDIS DEVELOPMENT AWARD CONSORTIUM members:** Athanasios Saratzis ^1^, Alun Davies ^2^, Sarah Onida ^2^, Rachel Phillips ^3^, Shaun Barber ^4^, Nicolas Johnson ^3^, Thomas Withers ^5^, Laura Burgess ^2^, Rebecca Lawton ^2^, Sarah Whittley ^2^, Colin Bicknell ^2^, Matt Bown ^6^, David Epstein ^7^, Justin Waring ^8^, David Wingfield ^2^, Colin Greaves ^5^, Lindsay Bearne ^9^, Matthew Popplewell ^10^, Matthew Machin ^2^, Chris Twine ^11^, Amy Harwood ^12^, George Smith ^13^, Maciej Juszczak ^14^, Faye Ashton ^15^, Athanasios Diamantopoulos ^16^, Mohamad Hamady ^17^, Patrick Coughlin ^18^, Prakash Saha ^19^, Smaragda Lampridou ^2^, Pasha Normahani ^2^, Steven Rogers ^20^, Konstantinos Stavroulakis ^21^, Isabelle Van Herzeele ^22^, Leonard Shan ^23^, Elizabeth Broadbent ^24^, Manar Khashram ^25^, Oliver Lyons ^26^, Ruth Benson ^27^, John Norrie ^28^, Andrew Stoddart ^28^, Babak Choodari-Oskooei ^29^, Max Parmar ^29^, Marcus Jepson ^30^, Barnaby Carter ^5^, Natalia Klimowska-Nassar ^3^, Ed Waddingham ^3^, Catherine Sudlow ^31^, Gordon Moran ^32^, Colin Crooks ^32^, Jugdeep Dhesi ^33^, Terry Tynski ^34^

Affiliations of the PAEDIS DEVELOPMENT AWARD CONSORTIUM members:

^1^ Cardiovascular Sciences, University of Leicester, Glenfield Hospital, Leicester, Leicestershire, LE39QP, UK

^2^ Section of Vascular Surgery, Department of Surgery and Cancer, Imperial College London, London, UK

^3^ Imperial Clinical Trials Unit, Imperial College London, UK

^4^ Leicester Clinical Trials Unit, University of Leicester, UK

^5^ School of Sport, Exercise and Rehabilitation Sciences, University of Birmingham, UK

^6^ Department of Vascular Surgery, University of Leicester, UK

^7^ Department of Applied Economics, University of Granada, Spain

^8^ Health Services Management Centre, University of Birmingham, UK

^9^ Population Health, Research Institute, St George's, University of London, UK

^10^ Department of Vascular Surgery, University of Birmingham, UK

^11^ Department of Vascular Surgery, North Bristol NHS Trust, UK

^12^ Sport and Exercise Sciences, Manchester Metropolitan University, UK

^13^ Department of Vascular Surgery, Hull University Teaching Hospitals NHS Trust, UK

^14^ Department of Vascular Surgery, University Hospitals Birmingham NHS Foundation Trust, UK

^15^ Department of Vascular Surgery, University Hospitals of Leicester NHS Trust, UK

^16^ Department of Interventional Radiology, Guy's and St Thomas' NHS Foundation Trust, UK

^17^ Department of Surgery & Cancer, Imperial College Healthcare NHS Trust, UK

^18^ Department of Vascular Surgery, Cambridge University Hospitals NHS Foundation Trust, UK

^19^ Department of Vascular Surgery, Guy's and St Thomas' NHS Foundation Trust, UK

^20^ Division of Cardiovascular Sciences, The University of Manchester, UK

^21^ Department of Vascular Surgery, Ludwig Maximillian University of Munich, Germany

^22^ Department of Thoracic and Vascular Surgery, Ghent University, Belgium

^23^ Department of Surgery, The University of Melbourne, Australia

^24^ Department of Psychological Medicine, The University of Auckland, New Zealand

^25^ Department of Vascular Surgery, The University of Auckland, New Zealand

^26^ Department of Surgery, Canterbury District Health Board, New Zealand

^27^ Department of Vascular Surgery, Canterbury District Health Board, New Zealand

^28^ Edinburgh Clinical Trials Unit, The University of Edinburgh, UK

^29^ MRC Clinical Trials Unit, University College London, UK

^30^ Bristol Medical School, University of Bristol, UK

^31^ Data Science Centre, The University of Edinburgh, UK

^32^ Faculty of Medicine & Health Sciences, The University of Nottingham, UK

^33^ Department of Geriatric Medicine, Guy's and St Thomas' NHS Foundation Trust, UK

^34^ Patient co-investigator, Glasgow, UK

^35^ Patient co-investigator, London, UK

^36^ Patient co-investigator, Leicester, UK

## References

[1] Saratzis A Jaspers NEM Gwilym B : Observational study of the medical management of atients with Peripheral Artery Disease. *Br J Surg.* 2019;106(9):1168–1177. 10.1002/bjs.11214 31259387

[2] Horvath L Nemeth N Feher G : Epidemiology of Peripheral Artery Disease: narrative review. *Life (Basel).* 2022;12(7):1041. 10.3390/life12071041 35888129 PMC9320565

[3] Kyle D Boylan L Wilson L : Accuracy of Peripheral Artery Disease registers in UK general practice: case-control study. *J Prim Care Community Health.* 2020;11: 2150132720946148. 10.1177/2150132720946148 32959726 PMC7513392

[4] Conte MS Bradbury AW Kolh P : Global vascular guidelines on the management of chronic limb-threatening ischemia. *Eur J Vasc Endovasc Surg* 2019;58(1S):S1–S109. e33. 10.1016/j.ejvs.2019.05.006 31182334 PMC8369495

[5] Criqui MH Aboyans V : Epidemiology of peripheral artery disease. *Circ Res.* 2015;116(9):1509–26. 10.1161/CIRCRESAHA.116.303849 25908725

[6] Le Boutillier C Saratzis A Saha P : Factors that influence the feasibility and implementation of a complex intervention to improve the treatment of peripheral arterial disease in primary and secondary care: a qualitative exploration of patient and provider perspectives. *BMJ Open.* 2023;13(1): e066883. 10.1136/bmjopen-2022-066883 36690397 PMC9872459

[7] Wong KHF Zlatanovic P Bosanquet DC : Antithrombotic therapy for aortic aneurysms: a systematic review and meta-analysis. *Eur J Vasc Endovasc Surg.* 2022;64(5):544–556. 10.1016/j.ejvs.2022.07.008 35853579

[8] Salem M Hosny MS Francia F : Management of extensive Aorto-Iliac disease: a systematic review and meta-analysis of 9319 patients. *Cardiovasc Intervent Radiol.* 2021;44(10):1518–35. 10.1007/s00270-021-02785-6 34279686

[9] Saratzis A Lea T Yap T : Paclitaxel and mortality following peripheral angioplasty: an adjusted and case matched multicentre analysis. *Eur J Vasc Endovasc Surg.* 2020;60(2):220–229. 10.1016/j.ejvs.2020.04.008 32370918

[10] Saratzis A Paraskevopoulos I Patel S : Supervised Exercise Therapy and revascularization for intermittent claudication: network meta-analysis of randomized controlled trials. *JACC Cardiovasc Interv.* 2019;12(12):1125–36. 10.1016/j.jcin.2019.02.018 31153838

[11] Twine CP Kakkos SK Aboyans V : Editor's choice - European Society for Vascular Surgery (ESVS) 2023 clinical practice guidelines on antithrombotic therapy for vascular diseases. *Eur J Vasc Endovasc Surg.* 2023;65(5):627–89. 10.1016/j.ejvs.2023.03.042 37019274

[12] Troisi N Saratzis A Katsogridakis E : Different Endovascular Modalities of treatment for isolated atherosclerotic Popliteal artery lesions (EMO-POP) registry. *J Vasc Surg.* 2023;77(1):231–240. e4. 10.1016/j.jvs.2022.07.170 35934215

[13] Watson E Bridgwood B Saha P : A Community and Hospital cAre Bundle to improve the medical treatment of severe cLaudIcation and critical limb iSchaemia (CHABLIS) [version 1; peer review: 2 approved]. *NIHR Open Res.* 2022;2:58. 10.3310/nihropenres.13341.1 37881303 PMC10593312

[14] Kaneta G Husain S Musto L : Eligibility of Common Femoral Artery atherosclerotic disease for endovascular treatment - the CONFESS study. *Eur J Vasc Endovasc Surg.* 2022;64(6):684–691. 10.1016/j.ejvs.2022.08.034 36075540

[15] Saratzis A Stavroulakis K : Contemporary endovascular management of Common Femoral Artery atherosclerotic disease. *Br J Surg.* 2021;108(8):882–884. 10.1093/bjs/znab150 33984124 PMC10364864

[16] Saratzis A Bosanquet DC Benson RA : Current medical management of patients with Peripheral Arterial Disease and potential benefits of risk-factor optimization: a Vascular and Endovascular Research Network (VERN) collaboration. *Eur J Vasc Endovasc Surg.* 2020;59(5):e33. 10.1016/j.ejvs.2019.12.015

[17] Saratzis A Jaspers NEM Gwilym B : Observational study of the medical management of patients with peripheral artery disease. *Br J Surg.* 2019;106(9):1168–1177. 10.1002/bjs.11214 31259387

[18] Bradbury AW Moakes CA Popplewell M : A vein bypass first versus a best endovascular treatment first revascularisation strategy for patients with chronic limb threatening ischaemia who required an infra-popliteal, with or without an additional more proximal infra-inguinal revascularisation procedure to restore limb perfusion (BASIL-2): an open-label, randomised, multicentre, phase 3 trial. *Lancet.* 2023;401(10390):1798–809. 10.1016/S0140-6736(23)00462-2 37116524

[19] Menard MT Rosenfield K Farber A : The BEST-CLI trial: implications of the primary results. *Eur J Vasc Endovasc Surg.* 2023;65(3):317–9. 10.1016/j.ejvs.2022.12.032 36621707

[20] Farber A Menard MT Conte MS : Surgery or endovascular therapy for Chronic Limb-Threatening Ischemia. *N Engl J Med.* 2022;387(25):2305–16. 10.1056/NEJMoa2207899 36342173

[21] Lyons OT Behrendt CA Bjorck M : Beyond wires and knives: what can we learn from BEST-CLI and BASIL-2? *Eur J Vasc Endovasc Surg.* 2023;66(1):1–3. 10.1016/j.ejvs.2023.05.032 37217073

[22] Anand SS Hiatt W Dyal L : Low-dose rivaroxaban and aspirin among patients with Peripheral Artery Disease: a meta-analysis of the COMPASS and VOYAGER trials. *Eur J Prev Cardiol.* 2022;29(5):e181–e9. 10.1093/eurjpc/zwab128 34463737

[23] Eikelboom JW Connolly SJ Bosch J : Rivaroxaban with or without aspirin in stable cardiovascular disease. *N Engl J Med.* 2017;377(14):1319–30. 10.1056/NEJMoa1709118 28844192

[24] Bearne L Galea Holmes M Bieles J : Motivating Structured walking Activity in people with Intermittent Claudication (MOSAIC): protocol for a randomised controlled trial of a physiotherapist-led, behavioural change intervention versus usual care in adults with intermittent claudication. *BMJ Open.* 2019;9(8): e030002. 10.1136/bmjopen-2019-030002 31446416 PMC6720323

[25] Bearne LM Volkmer B Peacock J : Effect of a home-based, walking exercise behavior change intervention vs usual care on walking in adults with peripheral artery disease: the MOSAIC randomized clinical trial. *JAMA.* 2022;327(14):1344–55. 10.1001/jama.2022.3391 35412564 PMC9006109

[26] Guide to the methods of technology appraisal 2013. *NICE Process and Methods Guides.* London,2013. Reference Source

[27] Bhadhuri A Kind P Salari P : Measurement properties of EQ-5D-3L and EQ-5D-5L in recording self-reported health status in older patients with substantial multimorbidity and polypharmacy. *Health Qual Life Outcomes.* 2020;18(1): 317. 10.1186/s12955-020-01564-0 32993637 PMC7526382

[28] Walters SJ Brazier JE : Comparison of the minimally important difference for two health state utility measures: EQ-5D and SF-6D. *Qual Life Res.* 2005;14(6):1523–32. 10.1007/s11136-004-7713-0 16110932

[29] Meecham L Popplewell M Bate G : Comparison of Femoropopliteal Plain Balloon Angioplasty for chronic limb-threatening ischemia in the BASIL trial and in a UK contemporary series. *J Vasc Surg.* 2021;74(6):1948–55. 10.1016/j.jvs.2021.06.475 34298121

[30] Farber A Menard MT Conte MS : Surgery or endovascular therapy for Chronic Limb-Threatening Ischemia. *N Engl J Med.* 2022;387(25):2305–2316. 10.1056/NEJMoa2207899 36342173

[31] Saratzis A Paraskevopoulos I Patel S : Supervised Exercise Therapy and Revascularization for Intermittent Claudication: Network meta-analysis of randomized controlled trials. *JACC Cardiovasc Interv.* 2019;12(12):1125–1136. 10.1016/j.jcin.2019.02.018 31153838

[32] Boufi M Ejargue M Gaye M : Systematic review and meta-analysis of endovascular versus open repair for Common Femoral Artery atherosclerosis treatment. *J Vasc Surg.* 2021;73(4):1445–55. 10.1016/j.jvs.2020.10.026 33098944

[33] Fong KY Xin L Ng J : A systematic review and meta-analysis of sirolimus-eluting stents for treatment of below-the-knee arterial disease. *J Vasc Surg.* 2023;77(4):1264–73. e3. 10.1016/j.jvs.2022.09.022 36183989

[34] Katsanos K Spiliopoulos S Teichgraber U : Editor's choice - risk of major amputation following application of paclitaxel coated balloons in the lower limb arteries: a systematic review and meta-analysis of Randomised Controlled Trials. *Eur J Vasc Endovasc Surg.* 2022;63(1):60–71. 10.1016/j.ejvs.2021.05.027 34326002

[35] Speich B von Niederhausern B Schur N : Systematic review on costs and resource use of Randomized Clinical Trials shows a lack of transparent and comprehensive data. *J Clin Epidemiol.* 2018;96:1–11. 10.1016/j.jclinepi.2017.12.018 29288136

[36] Stavroulakis K Katsogridakis E Torsello G : Editor's choice - RANDOMisation screening for drug coated or drug eluting device randomised trials among patients undergoing endovascular femorOPopliteal procedures (RANDOM-STOP study). *Eur J Vasc Endovasc Surg.* 2023;66(3):362–8. 10.1016/j.ejvs.2023.06.038 37406876

