# Supplementary material for: Designing a platform/adaptive randomised controlled trial for peripheral arterial disease (PAD) – The PAEDIS international platform trial development project
Source: NIHR Open Res. 2024 Apr 29;4:24. [Version 1] doi: 10.3310/nihropenres.13556.1 (PMC11391191; doi:10.3310/nihropenres.13556.1)
Supplement: Peer review history

# Reviewer report: Reviewer response for version 1

**DOI:** 10.3310/nihropenres.14715.r32243

**Published:** 2024-09-24

**Authors:** Harri Hakovirta

**Affiliations:** 1 University of Turku, Turku, Finland

The present study protocol accesses a challenging, important topic -treatment. As stated many basic issues like follow-up and treatment approach of most common clinical presentations of PAD still remain controversial and the strong scientific base is lacking.

 The study protocol is all written and extensive.

 The main comments concern definitions. The patient group in the workshops would benefit more detailed description and inclusion, exclusion criteria. How many with claudication, CLTI...

 Also the study design might be more accurate if the possible multilevel disease would be considered for CLTI patients. In common practice pure crural or SFA disease is not that common and many have high burden of atherosclerosis involving even all segments of lover limb arteries.

 It is not clear if patient with significant SFA and crural vessel stenosis would fall into crural or SFA cohort. Also no- significant stenosis at several other segments than to be treated might have impact on 2-year outcome.

 As authors state the clinical setting for the study question is challenging. The setting of 4 conservative treatments with claudication is clear since all claudicants are included. Also for the CLTI endovascular versus open with or without  follow-up is clear and relevant.

 It would be nice to add also non-invasive pressure measurements for study to have the baseline level for how severe arterial insufficiency preceeded the selected treatment and if treatment had any positive  short term input on peripheral circulation.

Is the study design appropriate for the research question?

Yes

Is the rationale for, and objectives of, the study clearly described?

Yes

Are sufficient details of the methods provided to allow replication by others?

Partly

Are the datasets clearly presented in a useable and accessible format?

Yes

Reviewer Expertise:

Peripheral artery disease

I confirm that I have read this submission and believe that I have an appropriate level of expertise to confirm that it is of an acceptable scientific standard.

# Reviewer report: Reviewer response for version 1

**DOI:** 10.3310/nihropenres.14715.r31723

**Published:** 2024-09-11

**Authors:** Raul J Guzman

**Affiliations:** 1 Yale University, New Haven, Connecticut, USA

The authors describe a platform randomized trial protocol for assessment of various interventions in patients with PAD. They appropriately divide PAD patients into 2 cohorts, those with intermittent claudication and those with chronic limb threatening ischemia. The protocols were developed over 11 months through workshops involving 44 patients with PAD and 112 experts. The proposed platform involves two multi-arm, multi-stage randomized studies. This is an **excellent outline **for initiation of a platform study designed to assess various treatment strategies for patients with ischemic claudication and CLTI.

 Many protocol details will need to be developed such as how the diagnosis of IC will be confirmed (pre-post exercise ABIs vs clinical criteria), definition of CLTI, and related inclusion/exclusion criteria. It also will be important to divide patients with CLTI into those with rest pain and non-healing ulcers.  What is the extent of foot ulceration allowable? Include calcaneal ulcers? What objective criteria will be used to make sure comparable patients are recruited across all sites?  

 Unclear what is meant by anticipated recruitment of 0.84 CFA and 0.38 BTK participants monthly. Please explain.

 Platform trials typically envision the serial testing of future technologies or treatments. Are there any other treatments being considered for future investigation?

Is the study design appropriate for the research question?

Yes

Is the rationale for, and objectives of, the study clearly described?

Yes

Are sufficient details of the methods provided to allow replication by others?

Yes

Are the datasets clearly presented in a useable and accessible format?

Not applicable

Reviewer Expertise:

PAD, arterial calcificaiton

I confirm that I have read this submission and believe that I have an appropriate level of expertise to confirm that it is of an acceptable scientific standard.

# Reviewer report: Reviewer response for version 1

**DOI:** 10.3310/nihropenres.14715.r32520

**Published:** 2024-08-26

**Authors:** Eric Secemsky

**Affiliations:** 1 Harvard Medical School, Boston, Massachusetts, USA

Professor Saratzis and colleagues have submitted the protocol outlining the PAEDIS platform for vascular trials to answer remaining question in interventional treatment of PAD. This is an incredibly thorough, meticulous approach to developing a flexible trial platform to advance the knowledge of treatment care for PAD. 

 It would be helpful to understand better how participants were selected and diversity in the participants was adequate. In particular, it appears the BSIR was involved but ensuring there was adequate representation of non-surgical proceduralists is key and can be better identified in the text.

 The identified metrics in the proposed complex PAD trial are commendable but worth discussion. Both COMPASS and VOYAGER answered critical questions about Riva in PAD. Voyager included patients who were undergoing surgical revasc, so more clear explanations of the need for additional trials would be helpful. In addition, the outcomes for this study would be more focused on reductions in CV and limb related events  (ALI, repeat revasc) over quality of life. 

 The other trials are well designed and important questions to answer. I always have concerns with the best approach for assessing compliance with exercise therapy, in particular home based treatment. Hopefully there is a good way to assess this in the current trial proposal. 

 Overall this is a wonderful PAD platform design and important for improving the evidence base for vascular patients. This is a clean protocol and the methodology is easy to follow and the reasoning for these studies are sound.

Is the study design appropriate for the research question?

Yes

Is the rationale for, and objectives of, the study clearly described?

Yes

Are sufficient details of the methods provided to allow replication by others?

Yes

Are the datasets clearly presented in a useable and accessible format?

Yes

Reviewer Expertise:

Vascular intervention; medical therapy

I confirm that I have read this submission and believe that I have an appropriate level of expertise to confirm that it is of an acceptable scientific standard.

# Reviewer report: Reviewer response for version 1

**DOI:** 10.3310/nihropenres.14715.r32248

**Published:** 2024-07-24

**Authors:** Mohamad A Hussain, Abena Appah-Sampong

**Affiliations:** 1 Brigham and Women's Hospital, Massachusetts, USA; 2 Surgery, Brigham and Women's Hospital, Boston, Massachusetts, USA

***Summary:*** *The study proposes the design of a platform randomized control trial to the assess safety, long term outcomes, and cost effectiveness of several interventions for symptomatic PAD that are currently in clinical use without substantial evidence.  The authors present a very detailed and thoughtful design methodology that was built in five stages that the authors call "work packages." These packages demonstrate a thorough review of the vascular disease literature to inform the study design, creation of research groups that is inclusive of international clinical experts on PAD and patients, and a collaborative and iterative process to finalize the trial protocol.* *The study review identifies gaps in the literature assessing effectiveness of new invasive and pharmacologic PAD treatments including optimal exercise therapy strategy, optimal anticoagulation strategies prior to surgery, efficacy of endovascular intervention of CFA and stenting of crural arteries, and optimal ultrasound surveillance strategies after endovascular intervention for symptomatic PAD.*

***Major Comment:*** *The proposed trial program is large and complex with high costs. Consider publishing detailed protocols for the intended subprojects that may serve useful for investigators to use as a framework to execute substudies.*

Is the study design appropriate for the research question?

Partly

Is the rationale for, and objectives of, the study clearly described?

Yes

Are sufficient details of the methods provided to allow replication by others?

Partly

Are the datasets clearly presented in a useable and accessible format?

Not applicable

Reviewer Expertise:

Clinical Epidemiology & Health Services Research

We confirm that we have read this submission and believe that we have an appropriate level of expertise to confirm that it is of an acceptable scientific standard, however we have significant reservations, as outlined above.

# Response

**Published:** 2024-08-27

**Authors:** Athanasios Saratzis

This work has developed a study design and protocol structure for a potential future platform/adaptive randomised controlled trial relating to peripheral artery disease. It can be used as a blueprint for future trials in this area.

 Exhaustive protocols for each component/package of the proposed work cannot be published at this stage, as the final trial design has not been funded by the NIHR. As a result, we have not included the detailed design of each proposed trial component +/- a full protocol, as this was beyond the scope of the funded developmental work. This work should act as a basis for future trial designs and is not intended to act as a finalised protocol for a study/project.
